# Endogenous social distancing and its underappreciated impact on the epidemic curve

**DOI:** 10.1038/s41598-021-82770-8

**Published:** 2021-02-04

**Authors:** Marko Gosak, Moritz U. G. Kraemer, Heinrich H. Nax, Matjaž Perc, Bary S. R. Pradelski

**Affiliations:** 1 Faculty of Natural Sciences and Mathematics, University of Maribor, Koroška cesta 160, 2000 Maribor, Slovenia; 2 Faculty od Medicine, University of Maribor, Taborska ulica 8, 2000 Maribor, Slovenia; 3 Department of Zoology, University of Oxford, Mansfield Road, Oxford, OX1 3SZ UK; 4 Harvard Medical School, 25 Shattuck St, Boston, 02115 USA; 5 Behavioral Game Theory, ETH Zurich, Clausiusstrasse 37, 8092 Zurich, Switzerland; 6 Institute of Sociology, University of Zurich, Andreasstrasse 15, 8050 Zurich, Switzerland; 7 Department of Medical Research, China Medical University Hospital, China Medical University, Taichung, Taiwan; 8 Complexity Science Hub Vienna, Josefstädterstraße 39, 1080 Vienna, Austria; 9 Univ. Grenoble Alpes, CNRS, Inria, Grenoble INP, LIG, 38000 Grenoble, France

**Keywords:** Computational science, Statistical physics, thermodynamics and nonlinear dynamics

## Abstract

Social distancing is an effective strategy to mitigate the impact of infectious diseases. If sick or healthy, or both, predominantly socially distance, the epidemic curve flattens. Contact reductions may occur for different reasons during a pandemic including health-related mobility loss (severity of symptoms), duty of care for a member of a high-risk group, and forced quarantine. Other decisions to reduce contacts are of a more voluntary nature. In particular, sick people reduce contacts consciously to avoid infecting others, and healthy individuals reduce contacts in order to stay healthy. We use game theory to formalize the interaction of voluntary social distancing in a partially infected population. This improves the behavioral micro-foundations of epidemiological models, and predicts differential social distancing rates dependent on health status. The model’s key predictions in terms of comparative statics are derived, which concern changes and interactions between social distancing behaviors of sick and healthy. We fit the relevant parameters for endogenous social distancing to an epidemiological model with evidence from influenza waves to provide a benchmark for an epidemic curve with endogenous social distancing. Our results suggest that spreading similar in peak and case numbers to what partial immobilization of the population produces, yet quicker to pass, could occur endogenously. Going forward, eventual social distancing orders and lockdown policies should be benchmarked against more realistic epidemic models that take endogenous social distancing into account, rather than be driven by static, and therefore unrealistic, estimates for social mixing that intrinsically overestimate spreading.

## Introduction

The contact rates of infectious and non-infectious agents play a key role in determining the epidemic curve. How effective policies for (partially or fully) voluntary contact reductions—such as social distancing recommendations, or orders without monitoring or penalty—are depends on individual decisions in the population.

The current COVID-19 pandemic illustrates that governments disagree quite fundamentally regarding how much freedom of choice their citizens can or ought to be entrusted with to achieve desirable levels of contact reductions, resulting in less (e.g. Sweden^1^) and more (e.g. China^2^) stringent policies. To identify adequate policy responses for a given population, it is important to understand both, how it will react during a pandemic, and how it will react to policies being introduced, that is, to understand endogenous *behavioral change*^3^? in order to formulate relevant epidemiological models.

Unfortunately, the interactive nature of behavioral change related to contact reductions and social distancing within a population during an infectious disease outbreak (compared with vaccination^4–7^, antiviral prophylaxis^8^, and travel^9^) has not been explored in much detail, in particular not regarding the individual-level game-theoretic foundations of social distancing, and how these compare with real-world evidence. Progress in this direction ought to be made, because game-theoretic analyses have shown that interactions can crucially shape the epidemic curve^10–13^, and modeling increasingly rests on rich assumptions regarding how individual behavior changes dynamically with the disease outbreak.

Making this step is particularly timely in light of the ongoing COVID-19 modeling, which has been highly policy-relevant. Currently, assumptions in these simulation models regarding behavioral change have to be made without adequate empirical foundations. The early simulations “driving the world’s response to COVID-19”^14^, for example, were based on static estimates of social mixing. These produced extreme scenarios in terms peak and case numbers of the outbreak, which were instrumental in governments’ justifications of policy introductions including of social distancing orders and lockdowns, in particular in the UK, but also across (most of) the globe. Recommending which policy options would be best suited to prevent these extreme scenarios, (many of the same) modelers again had to make relatively arbitrary behavioral assumptions regarding how social distancing policies would be adopted in terms of contact reductions in the population—as this example for the evaluation of a ‘voluntary home quarantine’ policy from^15^ illustrates: “Following identification of a symptomatic case in the household, all household members remain at home for 14 days. Household contact rates double during this quarantine period, contacts in the community reduce by 75%. Assume 50% of household comply with the policy”.

The exact numbers that are being assumed for comparative policy evaluations can obviously matter crucially. Hence, modeling such^15^, which currently is extremely policy-relevant, must move from making arbitrary assumptions to theoretically and empirically validated ones as soon as possible. Here, by integrating behavioral micro-foundations we make such a step, and draw on game theory to embed interactive decision-making of social distancing in an epidemiological model.

Many countries during the current pandemic have decided to introduce temporal restrictions on movement, contacts and social interactions, because the general impression was that voluntary social distancing recommendations would not be sufficient^16^. As economic and social costs of the pandemic and of the measures accumulate, governments increasingly consider restricting contacts less and more efficiently, that is, locally^17^ and/or dependent on health and risk status instead of locking down the whole population^18^. The issue is that research has not yet provided empirical benchmarks for endogenous contact rates in disease scenarios, so it is unclear how such policies can be evaluated scientifically: ideally a policy is benchmarked against a set of counterfactuals given the disease, not compared with what was before the disease. As such data is not available, we believe richer theory is needed, in particular game theory to provide a better rationale for making certain assumptions. We propose such a model, and to validate its basic logic compare its predictions with observations of contact rates during two influenza seasons in the United Kingdom where human contact was not affected by any noteworthy government policy. We view the observed effects as reasonable ‘lower bounds’ for counterfactual analyses in terms of what levels of social distancing ought to be expected from a more severe pandemic such as COVID-19 if no policies except educational ones were implemented. Our results suggest that levels of endogenous social distancing, as data suggests have been occurring during recent influenza seasons, might already flatten epidemic curves substantially. Policies need to be effective at least compared to such counterfactuals to warrant their introduction.

## Modeling infectious disease dynamics

The close monitoring and detailed modeling of infectious disease outbreaks has become an increasingly active research focus in epidemiology since the seminal works by Ref.^19–23^. The emergent body of research has substantially improved our understanding of infectious disease dynamics and how to control them (e.g. vaccination, quarantine, social distancing policies, etc.^24–26^), which together with the increasing availability of relevant data has allowed to apply some of these models to real-world epidemics.

One key modeling aspect concerns the transmission of infectious pathogens via individual contacts between infectious and susceptible individuals^27,28^, which have been shown to differ dependent on demographic factors such as age and sex^29–31^. While a lot of the prior work focuses on reconstructing the transmission trees of observed epidemics^32^, or on their final size and geographic spread^33,34^, less attention has been paid to the role that individual decision-making regarding social distancing—weighing the risks of infecting and being infected—plays in shaping behavioral contact patterns that underlie these dynamics.

Yet contact-seeking/avoiding decisions are key drivers of human disease dynamics. Descriptive analyses have revealed remarkable consistency at multiple temporal and spatial scales in the absence of exogenous factors^35–39^, and change as a result of disease severity^40^. One roadblock for making progress has been that relevant data are typically collected independently of health status. See^41^ for one of the first surveys conducted via telephone during an influenza season. While this is currently changing with growing numbers of health-tracking applications, there is no robust data that has been made available as of now. As a consequence, there is little empirical evidence on how human contact rates change depending on health status and as a function of disease incidence overall. Some related empirical work has been done to capture behavioral change in response to environmental disaster^42^, and travel restrictions^43,44^, but not in response to disease outbreaks except for some recent work that we shall discuss separately in the concluding remarks. Precisely this kind of insight, however, would be important to understand contact rate decisions, because the incentives to practice social distancing or not are different for healthy and for sick people. To improve predictions concerning the dynamics of diseases at the population level, and to understand what kinds of policies are actually appropriate, the next step is uncovering the behavioral determinants of contact patterns to better justify necessary assumptions.

A first rational-choice foundation of individual contact-seeking/avoiding behavior in response to an infectious disease in epidemiological models is a framework proposed by Fenichel et al.^45^. Contact rates of the resulting epidemiological model are no longer exogenous variables, but instead are determined co-evolutionarily with the dynamics of the disease itself. Their framework presents an individual risk assessment, presuming that individuals’ propensities to socially distancing increase with intensity and awareness of the disease due to the increased risks for contracting the disease. Simulations show that incorporating this type of individual decision-making changes predictions concerning the epidemic curve: flatter curves are the result, particularly if sick individuals also reduce their contacts.

The decision-theoretic framework by Ref.^45^ is a first step toward integrating human behavior into disease modeling, especially as regards understanding the role of infection fear. To improve the behavioral micro-foundations further, we proceed in two ways. First, the contact-reduction results are checked against some data on contact patterns during the 2012 and 2013 flu epidemics in the United Kingdom. Second, we account for the interactive nature of contact decisions by extending the underlying theoretical framework to a game-theoretic model. By using game theory we can model not just the trajectory of the disease as a function of the underlying contact data, but more generally endogenize contact patterns by an interactive decision model *and* as determined by the dynamics of the disease (e.g., incidence rates). Such a co-evolutionary view on contact rates and disease dynamics may substantially advance the resulting model’s predictive potential.

The core argument of this paper is that risk assessments underlying contact decisions are interactive, which we model by formulating the “social distancing game”. This game theory model permits us to produce testable individual-level comparative statics regarding how individuals will react during the outbreak of a disease and in response to others’ contact patterns (checking against data from two influenza seasons in the UK). Looking ahead, the advantage of our game-theoretic modeling approach is that it becomes feasible to identify tipping points in the underlying dynamics (as in^46,47^), whose transitions may be explosive and differ fundamentally for marginally different starting conditions as compared to those predicted by a non-game-theoretic model^48^. Future epidemic modeling should therefore consider game-theoretic modeling so as to leverage possible social dynamics of equilibrium transitions to policy advantage, as has been used in other policy domains^49,50^.

Two very recent concurrent papers make progress in this direction, and future work could merge our line of analyses with theirs. The first is^51^ which considers a theoretical model with endogenous contact rates where the two types of agents, sick (and infectious but not yet symptomatic) and healthy, who choose contact rates are in the same information set. Infected individuals stay at home with probability one. Their model generates the same contact rates for both types, and does not make predictions regarding interaction effects of the two. Our data indicates that health status leads to different contact patterns, and that symptomatic individuals also vary contact rates as a function of incidence. This is also an important feature of our simulations. A very nice feature the model in^51^, which points towards interesting avenues for future work, is an explicit treatment of the path dependency of equilibrium. The second paper is a related theoretical framework by Ref.^52^ who do not consider endogenous contact reductions by infected individuals at all because they have no private benefit from it. In that sense their model is more similar to^45^ than ours, but adds a Nash equilibrium analysis to it. Again, our data indicates that infected and infectious individuals do also reduce contact rates with incidence levels, and that there are interactions between contact rates of sick and healthy individuals. Pro-social concerns for the health of others, not just concerns for one’s own health, clearly play a very important role.

In sum, the ambition of this paper is to integrate behavioral responses from a game-theoretic framework into classical epidemiological models that accounts for health status and includes self-protective and pro-social concerns. By doing so, we propose a new model, spell out its behavioral predictions, in particular regarding differential rates of social distancing. We compare theoretical results with empirical observations from the 2012 and 2013 influenza epidemic in the United Kingdom, and discuss implications for policy recommendations in light of the simulated epidemic curves our model generates. We compare the endogenous curve with curves that would result from interventions such as immobilizing certain fractions of the population.

## Methods

### Contact rates

The key ingredients, implicitly behavioral ones, that determine the dynamics of epidemiological models are ‘contact rates’ which govern the frequency and likelihood of human interactions and therefore transmissions: *Where do you go? Who do you see? How do you make contact?* At the individual level, a change in contact rates may occur for symptom-specific medical reasons after contracting a disease that leads to reduced mobility for example. Moreover, a person, whether infected or not, may consciously decide to social distance, that is, to reduce contacts in light of various evolving risks (i.e. of spreading the disease and/or of contracting the disease) during an outbreak. Instead of using the social distancing terminology, we referred to social distancing as ‘(partial) self-quarantine/isolation’ in earlier versions of the paper, but adopt this jargon in line with^45^ as is becoming standard.

To understand the implications of these endogenous phenomena, we need a model for how and why behavioral change occurs during outbreaks of infectious diseases. To do so, we extend the existing decision-theoretic model of^45^ to allow for interactive decisions and *strategic* considerations as the risks of contracting and transmitting a disease depend on one’s own contact patterns as well as on everyone else’s level of social distancing. Therefore, we model the individual decision as dependent on others’ decisions, and we identify the rational-choice predictions for these decisions. By combining the human perspective on decision-making including considerations of risks and interactions in this way—using game theory—we obtain new and testable predictions for how human contact patterns and mobility decisions interact.

To illustrate the interactive nature of the proposed problem, consider the following thought experiments at the two extremes of the logical spectrum. At one extreme, suppose that everyone (sick and healthy alike) stays home, i.e. has reduced their contacts to zero (extreme social distancing). In that case, of course, any given individual (think of Will Smith as the only daytime person on the streets of NYC in “I Am Legend” to lighten the mood) can move freely without fear of infection (if healthy) or of infecting others (if sick). Thus, in game-theoretic language, this does not constitute a Nash equilibrium, because every individual prefers to deviate (from staying at home), given the decision of everyone else (to stay at home). At the other extreme, by contrast, when everyone (sick and healthy) is moving around all the time resulting in very high contact rates (no social distancing), it is safest to stay home in order to not become infected (if healthy) or not to infect others (if sick). Again, everyone moving freely around will not constitute an equilibrium.

### Social distancing in a population

In this section, we propose a formal model that will highlight the main advantages of choosing a game-theoretic rather than mechanistic approaches (as is done in applied work), and spell out how it goes beyond a single-player decision-theoretic model.

#### Population

Consider a human population $N=\{1,2,\ldots ,n\}$. Each person $i\in N$ either belongs to the set $H\subset N$, the *healthy* (or non-symptomatic, susceptible, uninfected, etc.), or to the other set $S=N{\setminus } H$, the *sick* (or symptomatic, non-susceptible, infected, etc.). Note that we work with a basic epidemic model setting without recovery (and repeat susceptibility) in mind, which naturally ought to be generalized in future work.

#### Social-distancing decisions

Each $i\in N$ chooses a contact rate $\beta _i\in [0,1]$. Write $\beta$ for the full vector of contact rates, $\beta _H$ for the average contact rate of healthy agents, and $\beta _S$ for the average contact rate of sick agents.

#### Utilities

Individual utility is generated by reaching places (or people) which is facilitated by being mobile. Hence, positive mobility is required to generate utility. But increased levels of mobility are also increasingly costly as they increase the exposure to infection risks for self and others. Hence, both complete immobility and full mobility generate no utility. Once there are risks of infection due to the presence of a disease, this mobility will be reduced to mitigate these risks.

Let us consider two scenarios distinguished by whether (1) everyone is healthy, or (2) there are infected individuals.

(1) *No-disease scenario* Suppose there is no disease, that is, $|S|=0$. In that case, we assume utility for any player *i* is described by a twice-differentiable, continuous utility function

1 $$\begin{aligned} {\mathbf{Base\, utility. }} \ \ \ u_{i}(\beta )= u(\beta _{i}) \end{aligned}$$

such that $u(0)=u(1)=0$, $u(\beta )>0$ for all $\beta \in (0,1)$, $u'(\beta )>0$ ($<0$) for $\beta <\beta ^{*}$ ($>\beta ^{*}$) given some $\beta ^{*}\in (0,1)$, and $u''(\beta )<0$. These assumptions ensure that $\beta ^{*}$ represents the unique utility-maximizing level of mobility in the no-disease scenario. We shall refer to levels chosen below $\beta ^{*}$ as ‘social distancing’. Of course, the optimal level will be heterogeneous within a population, but we abstract from this level of detail for the moment. Recent empirical work by Ref.^38,50^ identifies heterogeneous levels of mobility in the absence of a disease.

(2) *Disease scenario* Once some individuals are infected, that is, if $|S|>0$, then the ‘base utility’ $u(\beta _{i})$ that corresponds to a healthy individual, for an infected individual, is reduced directly by some disease factor $\delta$ (with $\delta \in [0,1]$ representing a proportional disutility from being sick) resulting in ‘sick utility’ $\delta u(\beta _{i})$. Moreover, depending on health status, all individuals suffer additional disutility from the risk of becoming infected (for healthy), or from the risk of infecting others (for sick), both of which increase with mobility, thus adding further costs to being mobile. Hence, for a **healthy individual**, $i \in H$ the utility is

2 $$\begin{aligned}&{\mathbf{Healthy }}\; H{\mathbf{-utility }} \ \ \ u_{i}(\beta ) =&\big (1-f\cdot \underbrace{\big (1-\big [\big (1-\beta ^{i}_{S}\big )^{n-|H|}\cdot \beta _{i} + 1-\beta _{i} \big ] }_{\text {infection risk}}\big )\big ) \ \ \cdot \underbrace{u(\beta _{i})}_{\text {base utility}} \end{aligned}$$

where $f\in [0,1]$ measures the fear of a healthy individual of getting infected, which also expresses disease severity. Similarly,

3 $$\begin{aligned}&{\mathbf{Sick }}\; S{\mathbf{-utility. }} \ \ \ u_{i}(\beta )=&\big (1-c\cdot \underbrace{\big (1-\big [\big (1-\beta ^{i}_{H}\big )^{|H|}\cdot \beta _{i}+1-\beta _{i}\big ]}_{\text {spreading risk}}\big )\big ) \ \ \cdot \underbrace{\delta u(\beta _{i})}_{\text {sick utility}} \end{aligned}$$

where $c\in [0,1]$ measures the pro-social concern an infected individual has for another individual’s life, that is, the expected reduction in utility from exposing other healthy humans to the risk of infection, which would naturally increase with the severity of the disease too. Note that the introduction of this parameter expressing this type of motivation, which is central to most policies aimed at reducing mobility of symptomatic humans, is absent in^45^, but will generate the kinds of mobility reductions that characterize several of his simulations resulting in the flattest epidemic curves.

The underlying contact scenario we thus express is one where $\beta _i$ represents agent *i*’s probability of exposing him/herself to an infection-risk encounter, and $1-(1-\beta ^{i}_{S})^{n-|H|}$ and $1-(1-\beta ^{i}_{H})^{|H|}$ respectively represent the probabilities of at least one infected/susceptible making the same encounter. Thus we model the probability of two parties meeting at a given location, or all parties spending some time at a central locations. W.l.o.g., when two individuals with different health status enter the location, we assume an infection takes place with probability one.

### Simulation details

We use random geometric graphs in hyperbolic spaces to generate networks that have heterogeneous degree distributions, strong clustering, and short average path lengths, which are all inherent properties of real social networks^53,54^. By increasing the curvature $\zeta$ of the hyperbolic space, we move from networks having exponential to networks having scale-free degree distributions, from longer to shorter average path lengths, and from weaker to stronger clustering. We thus cover the whole family of networks that are representative for real social networks^55^.

On top of these networks, we consider the susceptible-exposed-infectious-recovered (SEIR) model^56,57^, as used for describing the spreading of the COVID-19 disease^58^. Initially, we select $0.2\%$ of the nodes uniformly at random and designate them as infected (I). The remaining $99.8\%$ of the nodes are designated as susceptible (S). Moreover, every node *i* is assigned a contact rate $q_{i}$, where $q_{i}=0$ means the node is not exposed at all and thus has no way of becoming infected, while $q_{i}=1$ means the node is fully exposed to potentially become infected by all the other nodes to which it is connected. In terms of social distancing, we can think of $q_{i}$ as the factor multiplied to the optimal baseline level of contact-making behavior $\beta ^{*}$ from our model. $q_{i}=0$ means that node *i* is fully isolated, while $q_{i}=0.5$ means there is a $50\%$ chance node *i* will make contact with any other nodes to which it is connected, and $q_{i}=1$ means full contact-making behavior without social distancing as given by $\beta ^{*}$. We consider several models; the model without social distancing with $q_{i}=1$ for all nodes, the model with uniform social distancing such that $q_{i}<1$ for all nodes, the model with random social distancing such that a fraction *p* of nodes is selected at random and assigned $q_{i}=0.1$ instead of $q_{i}=1$, and, finally, the model motivated by our game-theoretic analysis with endogenous social distancing fitted against the Flusurvey data to account for decreasing $q_{i}$ as the fraction of infected individuals $\rho$ in the population increases. In particular, the function we use is $q_{i} =3^{(-10 \rho )}$, which yields a three-fold decrease in $q_{i}$ at 10% of infected in the population (which we approximate by extrapolating from the behavior of ill individuals from the observed values between 3 and 8% of infected in the population where we fitted such a slope—see top left of Fig. 3). We refer to the Supplementary Information files for details concerning the Monte Carlo method that we use to simulate this model including the code.

## Comparative statics

**Table 1:** Comparative statics of the equilibrium analysis. These describe how optimal contact rates vary with the other parameters, evaluated under the assumption that a symmetric Nash equilibrium exists such that $\beta _{i}=\beta _{H}$ for all healthy and $\beta _{i}=\beta _{S}$ for all sick. See Supplementary Information ‘Comparative Statics Derivations’ for details.

A marginal increase in ...	... leads to ...
Social distancing of healthy	Less social distancing of sick
Social distancing of sick	Less social distancing of healthy
Size of the healthy population	More social distancing of sick$^{!}$
Size of the sick population	More social distancing of healthy$^{!}$
Pro-social concern of the sick	More social distancing of sick
Dear of disease of the healthy	More social distancing of healthy*

*Present in the model by Ref.^45^. The other effects are new

$^{!}$Contrary to imitation, herding, etc. as proposed, for example, in^11,59^.

What interests us from the game-theoretic model are the comparative statics of contact rates in equilibrium when chosen optimally so as to maximize subjective expected utilities. These we obtain from the first-order conditions for optimal behavior for the two utility functions given by Eqs. (2) and (3), which we obtain by maximizing both expressions with respect to $\beta _i$:

4 $$\begin{aligned}&H{\mathbf{-FOC. }}\; {\overbrace{\big (1-f\cdot \big (1-\big [\big (1-\beta ^{i}_{S}\big )^{n-|H|}\cdot \beta _{i} + 1-\beta _{i} \big ]\big )\big ) u'(\beta _{i})}^{\text {marginal utility effect}}} \ \nonumber \\&\qquad \qquad { \ = \underbrace{f\cdot \big (1-\big (1-\beta ^{i}_{S}\big )^{n-|H|}\big ) u(\beta _{i})}_{\text {marginal infection risk effect}}} \end{aligned}$$

5 $$\begin{aligned}&S{\mathbf{-FOC. }}\ \ \  { \overbrace{ \big (1-c\cdot \big (1-\big [\big (1-\beta ^{i}_{H}\big )^{|H|}\cdot \beta _{i}+1-\beta _{i}\big ]\big )\big ) u'(\beta _{i})}^{\text {marginal utility effect}}\ } \nonumber \\&\qquad \qquad {\ = \underbrace{ c\cdot \big (1- \big (1-\beta ^{i}_{H}\big )^{|H|}\big ) u(\beta _{i})}_{\text {marginal spreading risk effect}}} \end{aligned}$$

Note that both right-hand sides of the latter equations are positive, indicating that both marginal utilities $u'(\beta _{i})$s must also be positive; i.e. that we now must obtain lower contact rates for both sick and healthy individuals (compared with the utility-maximizing level of mobility $\beta ^{*}$ from the no-disease scenario) in order for FOCs to be satisfied than in the no-disease benchmark. This means that both sick and health individuals will engage in some optimal level of ‘social distancing’, that is, choosing a lower equilibrium utility than $\beta ^{*}$ from the no-disease scenario. The comparative statics are summarized in Table 1.

**Figure 1:**
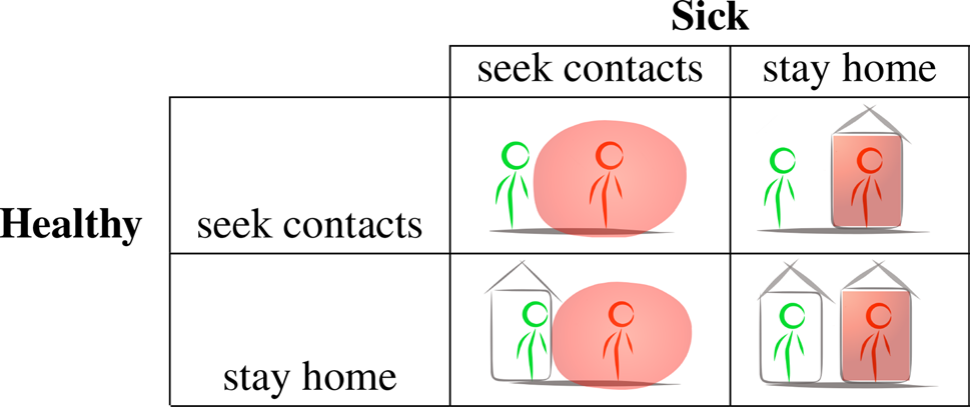
The social distancing interactions simplified.

Naturally, the optimal contact rates for healthy and sick are different and take intermediate values, the exact value depending on factors related to disease incidence, fear, concern, disease severity, risks, etc. Note that individuals may also differ in their fears, concerns, etc., hence we can think of the comparative statics in Table 1 also as organizing individual heterogeneity. While this is a two-population evolutionary game with continuous action space for every player, the strategic essence of this interaction can be represented by a simplified game played between Sick and Healthy as is illustrated in Fig. 1. Both health types seek contact leads to infection. Both staying home leads to no infection, but also generates zero utility for anyone. The two mixed outcomes, where only one party stays home, also do not lead to infection, and have the advantage that the population that continues to be mobile generates positive utilities. Similarly, if the elderly are particularly at risk, either the young or the elderly, or both, should perhaps avoid contacts with one another to avoid infections. As governments aim to return to higher levels of economic and social activities, such an outcome, with the sick (the high-risk groups) rather than the healthy rest reducing contacts, will likely become the goal.

Instead of assuming a particular functional form to obtain contact rates, we shall explore whether the direction of contact rate adjustments as per our comparative statics corresponds qualitatively with data from the UK Flusurvey (see Fig. 3). Flusurvey is a webtool managed and monitored by Public Health England (PHE). All experimental protocols were approved by the London School of Hygiene and Tropical Medicine Ethics Committee, and were carried out in accordance with relevant guidelines and regulations. Participation is voluntary, and informed consent was obtained from all subjects.

**Figure 2:**
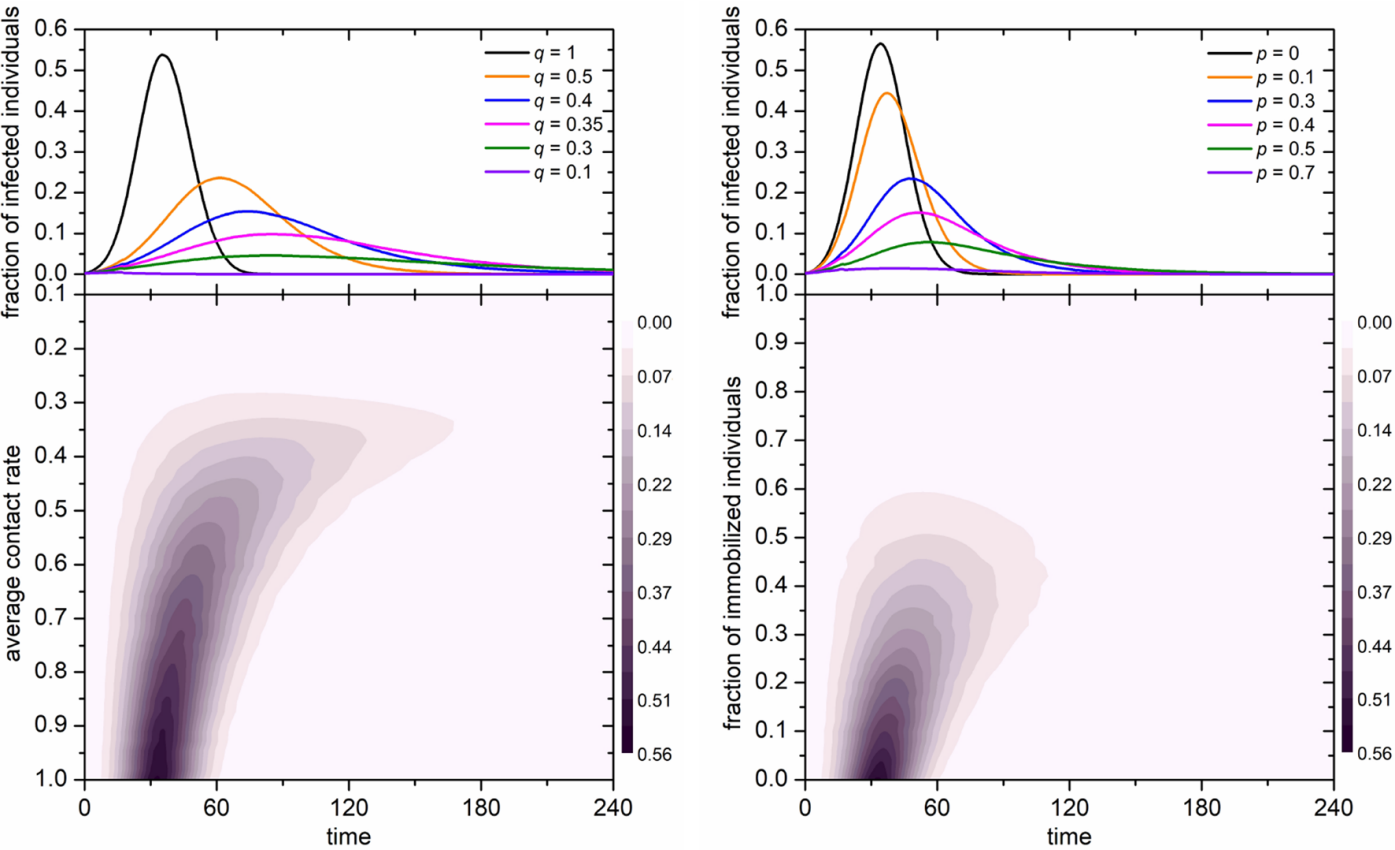
Simulated epidemic curves based on a susceptible-exposed-infectious-recovered (SEIR) model obtained by averaging outcomes over 1.5 million nodes in network configurations representing social networks (see the Simulation details section above and the Monte Carlo method section in Supplementary Information). Left: The color map encodes the fraction of infected individuals in dependence on time and the average contact rate. The upper panel shows characteristic cross-sections of the color map, where it can be observed that the endogenous contact reduction effect is matched no sooner than at 60–70% reduction of the contact rate (average mobility). Right: The color map encodes the fraction of infected individuals in dependence on time and the fraction of immobilized individuals. The upper panel shows characteristic cross-sections of the color map, where it can be observed that the endogenous contact reduction effect is matched no sooner than at 40–50% immobilization.

## Concluding remarks

*Who makes which contacts when?* is what needs to be modeled to obtain a realistic epidemic curve. An underappreciated element of contact behaviors is their interactive nature, and the cost-benefit analyses driving them. Health status, infection risk and behaviors of others matter for the individual decision in ways that state-of-the-art epidemiological modeling does not capture. In this paper, we proposed a rational-choice framework to endogenize these decisions and the resulting contact rates. While promising conceptually, a major issue for modeling behavior in general, and to test the kinds of predictions that our model generates in particular, is data availability. To date, very little data is available that records contact rates and health status at the same time. This might soon change as health-tracking applications are increasingly adopted.

**Figure 3:**
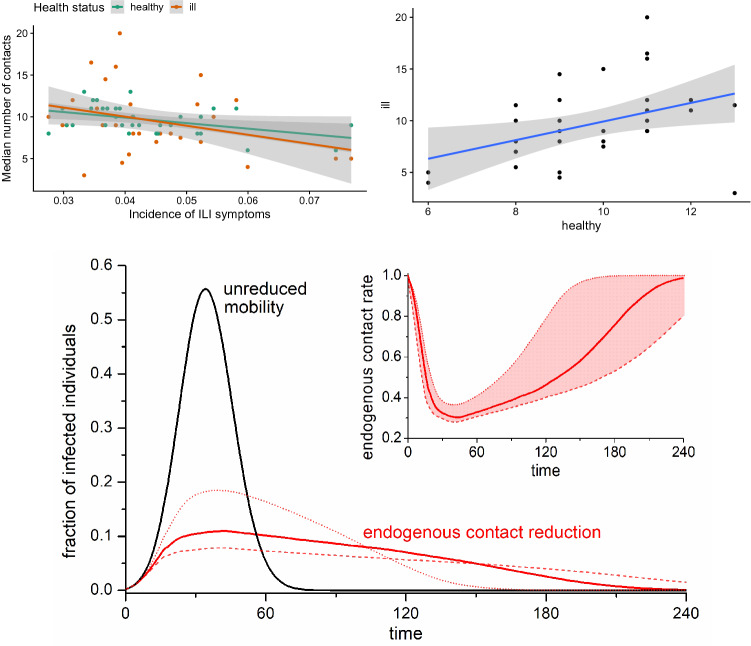
Social distancing for sick and healthy, and the effect on the epidemic curve. Top left: Median number of contacts in different weeks as a function of incidence of ILI symptoms among Flusurvey participants during that week. The lines show linear fits, and shades 95% confidence intervals. Slopes: healthy − 70 (95% CI − 120–(− 20)), ill − 110 (95 %CI − 220, 10); *p* value testing null hypothesis of slope 0: healthy 0.01, ill 0.07. Top right: Median number of contacts in participants with ILI symptoms as a function of the median number of contacts in participants without ILI symptoms. The line shows a linear fit, and shades 95% confidence intervals. Slope: 0.9 (95% CI 0.2–1.6); *p* value testing null hypothesis of slope 0: 0.02. Bottom: Comparison of the infected curves under unrestrained mobility (black) and endogenous contact reduction based on the Flusurvey data (black). The inset shows how fast the contact rate decreases as the fraction of infected individuals peaks, and then increases comparatively slowly as the incidence of infections decreases. Dashed and dotted lines were obtained with three-fold reductions at 5% and 15%, respectively, as the lower and upper bound on the error from the data (which suggest a three-fold decrease at about 9%/15% for ill/healthy—compare with slopes − 110/− 70 from top left). For a description of the model and simulations, see the Simulation details section above and the Monte Carlo method section in Supplementary Information.

As a first step, we simulated different interpretations of social distancing policies in Fig. 2, highlighting what kinds of epidemic curves ought to be expected from either reducing mobility of everyone in the population or from immobilizing a certain fraction. Towards some empirical foundations of these kinds of analyses, we considered Flusurvey data from the United Kingdom (see Fig. 3, and Supplementary Information ‘Influenza Contact Data’), where we found evidence of social distancing amongst healthy individuals as a function of disease incidences in their neighborhoods, as was predicted by our model and by earlier work. We pre-registered the basic notion of this kind of empirical analysis qualitatively before knowing what kind of data would be available exactly (see Open Science Framework projects osf.io/zc5b8 and osf.io/q3m2p). The baseline levels of contact making in the UK as recorded per Flusurvey (resulting in medians of circa 12–14 contacts per week outside the flu season) are in line with prior estimates from other countries than the UK^41,60^. Indeed, we found that, even in the context of seasonal influenza, some sizeable degree of social distancing took place amongst both sick (mobility reduction of ca. 50–55%) and healthy individuals (mobility reduction of ca. 30–35%), in line with endogenous social distancing at the population level in other countries^61^. We extrapolated these kinds of reductions linearly to levels outside the observed range in our simulations. Predicted negative correlations between the levels of the two health types were rejected, suggesting presence of behavioral elements beyond individual utility maximization such as social influence, norms, imitation, herding, etc.

We consider the fitting with influenza data useful, because a seasonal influenza virus is less severe than other pandemics such as SARS-CoV-2, so any endogenous mobility reductions observed for influenza would likely provide lower bounds on the reductions that we would expect (without policy) during a more severe pandemic such as the current one. In Sweden, for example, where the government decided against the kinds of lockdowns that other European countries implemented, the aggregate population mobility in transit and workplace decreased by 31% and 11% respectively between onset and peak of the pandemic (as per Google’s COVID-19 Community Mobility Report Sweden), which is comparable to the decrease we recorded in the Flusurvey. Our simulations in Fig. 3, which use the fit for contact reductions as observed in Flusurvey—extrapolating further reductions linearly in case of incidence levels beyond those observed, indicate that such reductions would flatten the epidemic curve to levels that are comparable in terms of height of the peak and total case numbers as would have been obtained from immobilizing 40–50% of the total population or bringing the average mobility down by 60–70%. These are candidate benchmarks we should be evaluating policy success against, not against historical data.

We are hopeful that future research and applied modeling will make use of game theory with the modeling framework we proposed. Further, we encourage future efforts to test our model’s hypotheses with more data and with data for COVID-19 instead of influenza, as there are potential confounding factors in our data related to the seasonality of contacts because of factors unrelated to disease (especially temperature, but also school holidays, etc.), which we cannot account for sufficiently due to data availability. Such analyses are important, as policymakers consider new, perhaps health-status dependent, mobility restrictions and relaxations thereof during the current pandemic. Ideally, to evaluate the effectiveness of such policies there ought to be at least some benchmarking concerning what levels might be expected endogenously, as well as monitoring of individual behaviors in response to changes in government recommendations or restrictions. For example, the UK COVID-19 lockdown from earlier this year is estimated to have reduced contacts by 75%^62^, which is roughly double the reduction we recorded for healthy individuals for the 2012 and 2013 influenza seasons (see Fig. 3), but not substantially above the levels that some of our simulations indicate would justify such policies (see Fig. 2). This work suggests scope for future studies in this directions and provides some first measurements.

Governments should factor in endogenous social distancing when weighing the pros and cons of policies as diverse as those ranging from China to Sweden. Epidemic modeling could improve its behavioral micro-foundations more generally.

## Supplementary information

Supplementary Information.

## Data Availability

The data underlying the analyses of this article is available upon request from Flusurvey.
