# Supplementary material for: Endogenous social distancing and its underappreciated impact on the epidemic curve
Source: Sci Rep. 2021 Feb 4;11:3093. doi: 10.1038/s41598-021-82770-8 (PMC7862686; doi:10.1038/s41598-021-82770-8)
Supplement: Supplementary file 1 — Supplementary Information. [file 41598_2021_82770_MOESM1_ESM.pdf]

Supplementary Information for ‘Endogenous social distancing and its underappreciated impact on the epidemic curve’

Marko Gosak[1,2], Moritz U. G. Kraemer[3,4], Heinrich H. Nax[5,6,*], Matjaž Perc[1,7,8], and Bary S. R. Pradelski[9]

[1]Faculty of Natural Sciences and Mathematics, University of Maribor, Koroška cesta 160, 2000 Maribor, Slovenia
[2]Faculty od Medicine, University of Maribor, Taborska ulica 8, 2000 Maribor, Slovenia
[3]Department of Zoology, Mansfield Road, University of Oxford, OX1 3SZ Oxford, UK
[4]Harvard Medical School, 25 Shattuck St, 02115 Boston, USA
[5]Behavioral Game Theory, Clausiusstrasse 37, ETH Zurich, 8092 Zurich, CH
[6]Institute of Sociology, Andreasstrasse 15, University of Zurich, 8050 Zurich, CH
[7]Department of Medical Research, China Medical University Hospital, China Medical University, Taichung, Taiwan
[8]Complexity Science Hub Vienna, Josefstädterstraße 39, 1080 Vienna, Austria
[9]Univ. Grenoble Alpes, CNRS, Inria, Grenoble INP, LIG, 38000 Grenoble, France
*heinrich.nax@uzh.ch

ABSTRACT

Influenza Contact Data

Data for **Fig. 3** comes from the UK Flusurvey (www.flusurvey.org.uk), an internet platform launched in 2009 to augment existing influenza surveillance[1,2]. The data underlying our analyses is available upon request from Flusurvey. Its focus is on recording healthcare usage by individuals with influenza-like-illness (ILI) symptoms[3,4]. During an influenza season, participants receive a weekly reminder to report presence or absence of ILI-related symptoms. When reported, followup questions are asked regarding health-care seeking and other behaviors. Flusurvey data has previously been used to estimate incidence trends[5], to identify risk factors[6], to estimate the effectiveness of vaccination[7], and to quantify health-care seeking behavior[8].

During the four influenza seasons 2009–13, social contact data were also collected, some of which is analyzed here. Participants were asked to report conversational and physical contacts by age group in three types of setting (home, work/school and other), as previously used to model H1N1v influenza[9]. Here, we use the total of conversational contacts reported as a proxy for overall contacts, and assessed whether the date at which the contacts were submitted were within the start and end dates of an episode of illness with ILI symptoms (one general symptom out of fever, tiredness, weakness and headache, and one respiratory symptom out of sore throat, cough and shortness of breath). The end date of an episode was considered to be a healthy date. We cleaned the data in the following ways. We removed bad symptom dates (end date before start date, dates after the date at which a response was submitted) in 85 out of 8800 symptom reports. We further removed all participants with fewer than three symptom reports (whether reporting healthy or ill), and removed the first submitted survey report of every participant in order to remove any potential bias from participants signing up only because they were researching influenza-related information. Where the end date of an episode was not reported, the date of the report which stated that the illness had ended was taken as the end date of the episode. Incidence was calculated as number of episodes of illness with ILI symptoms starting in any particular week divided by the number participants submitting a report in that week.

Data presented here are based on results from the UK flusurvey (www.flusurvey.org.uk), which was launched in 2009 as a platform for an internet-based cohort to augment existing influenza surveillance[1,2], most of which depends on recording healthcare usage by symptomatic individuals[3,4] and therefore misses individuals with influenza-like-illness (ILI) who do not seek medical attention. During the influenza season, every participants receives a weekly reminder via email, asking to report presence or absence of ILI-related symptoms. If such symptoms are reported, a number of followup questions are asked regarding health-care seeking and other behaviour on the previous day. The key questions of the Flusurvey relevant for this study ask, for ‘home’, ‘work’ and ‘other’, how many face-to-face and skin-to-skin contacts were made with people from age

groups 0-4, 5-18, 19-44, 45-64 and 65+ years respectively. Further relevant questions concern how much time was spent on public transport, in enclosed indoor spaces with more than 10 others, and what the furthest distance travelled from home was.

As well as estimating incidence trends[5], flusurvey data have been used to identify risk factors to ILI[6], to estimate the effectiveness of influenza vaccination[7] and to quantify health-care seeking behaviour[8]. During the four influenza seasons 2009–13, social contact data were collected in addition to the ILI-related data. Participants were asked to report conversational and physical contacts by age group in three types of setting (home, work/school and other). These data have previously been used to explain the spread of H1N1v influenza[9].

We used the total of conversational contacts reported as measure of overall contact, and assessed whether the date at which the contacts were submitted were within the start end end date of an episode of illness with ILI symptoms (one general symptom out of fever, tiredness, weakness and headache, and one respiratory symptom out of sore throat, cough and shortness of breath). The end date of an episode was considered to be a healthy date.

We cleaned the data in the following ways: We removed bad symptom dates (end date before start date, dates after the date at which a response was submitted) in 85 out of 8800 symptom reports. We further removed all participants with fewer than three symptom reports (whether reporting healthy or ill), and removed the first submitted survey report of every participant in order to remove any potential bias from participants signing up only because they were researching influenza-related information. Where the end date of an episode was not reported, the date of the report which stated that the illness had ended was taken as the end date of the episode.

Incidence was calculated as number of episodes of illness with ILI symptoms starting in any particular week divided by the number participants submitting a report in that week.

Monte Carlo Method

We perform Monte Carlo simulations of the SEIR model[10], which corresponds to a random sequential update, such that during a full Monte Carlo step (MCS) each node gets a chance once on average to become infected. Each full MCS consist of repeating the following elementary step n times. Firstly, select a node i uniformly at random from the whole network. Secondly, (i) If node i is in state S, choose one neighbor j uniformly at random and visit it with probability q_i. If the neighbor is visited and is in state I, node i becomes infected with probability $w = 0.7$. If, however, the neighbor j is in states S or R nothing happens. (ii) If node i is in state I, then verify if at least $t_r = 15$ full MCS have passed since it became infected. If yes, node i becomes recovered (R), and if no, node i remains infected. (iii) If node i is in state R, nothing happens.

Comparative Statics Derivations

We first rearrange H-FOC (Equation 2 in the main document):

$$
\begin{aligned}
& (1\text{-}f\cdot(1\text{-}[(1\text{-}\beta_S^i)^{n\text{-}|H|}\cdot\beta_i+1\text{-}\beta_i]))u'(\beta_i) \\
= \;& \text{-}f\cdot((1\text{-}\beta_S^i)^{n\text{-}|H|}\text{-}1)u(\beta_i) \\
\iff \;& (1+f\cdot(\text{-}1+[((1\text{-}\beta_S^i)^{n\text{-}|H|}\text{-}1)\cdot\beta_i+1]))u'(\beta_i) \\
= \;& \text{-}f\cdot((1\text{-}\beta_S^i)^{n\text{-}|H|}\text{-}1)u(\beta_i)
\end{aligned}
\tag{1}
$$

We are interested in the partial derivative of Equation 1 along d/df, d/dc, $d/d\beta_S$, and $d/d\beta_H$. As the calculations for the former two and the latter two are very similar we only detail them for d/df and $d/d\beta_S$. The other predictions also follow from similar arguments.

Partial derivative of Equation 1 along d/df:

$$
\begin{aligned}
& (1+f\cdot(-1+[((1-\beta_S^i)^{n-|H|}-1)\cdot\beta_i+1]))\cdot u''(\beta_i)\cdot\beta_i' \\
+ \;& u'(\beta_i)\left[-1+[((1-\beta_S^i)^{n-|H|}-1)\cdot\beta_i+1]\right] \\
- \;& u'(\beta_i)f\cdot[(1-\beta_S^i)^{n-|H|}-1]\cdot\beta_i' \\
= \;& -((1-\beta_S^i)^{n-|H|}-1)\cdot(f\cdot u'(\beta_i)\cdot\beta_i'+u(\beta_i)) \\
\iff \;& \beta_i'\cdot\Bigg[\overbrace{(1+f\cdot(-1+[((1-\beta_S^i)^{n-|H|}-1)\cdot\beta_i+1]))}^{>0}\cdot\overbrace{u''(\beta_i)}^{<0} \\
- \;& \overbrace{u'(\beta_i)\cdot f\cdot[(1-\beta_S^i)^{n-|H|}-1]}^{=(*)}+\overbrace{u'(\beta_i)\cdot f\cdot((1-\beta_S^i)^{n-|H|}-1)}^{=(*)}\Bigg] \\
= \;& -u'(\beta_i)\left[-1+[((1-\beta_S^i)^{n-|H|}-1)\cdot\beta_i+1]\right] \\
- \;& ((1-\beta_S^i)^{n-|H|}-1)\cdot u(\beta_i)
\end{aligned}
\tag{2}
$$

$$
\begin{aligned}
\Longleftrightarrow \quad & \beta_i' \cdot \Bigg[\overbrace{(1+f\cdot(-1+[((1-\beta_S^i)^{n-|H|}-1)\cdot\beta_i+1]))\cdot u''(\beta_i)}^{<0} \Bigg] \\
= \quad & \overbrace{u'(\beta_i)[-1+[((1-\beta_S^i)^{n-|H|}-1)\cdot\beta_i+1]]}^{>0} \\
+ \quad & \overbrace{(1-(1-\beta_S^i)^{n-|H|})\cdot u(\beta_i)}^{>0} \\
\Rightarrow \quad & \beta_i' < 0
\end{aligned}
$$

Partial derivative of Equation 1 along $d/d\beta_S$:

$$
\begin{aligned}
& (1+f\cdot(-1+[((1-\beta_S^i)^{n-|H|}-1)\cdot\beta_i+1]))\cdot u''(\beta_i)\cdot\beta_i' \\
+ \quad & u'(\beta_i)\cdot f\cdot \Bigg[((1-\beta_S^i)^{n-|H|}-1)\beta_i' - \beta_i(1-\beta_S^i)^{n-|H|-1} \Bigg] \\
= \quad & -f\cdot((1-\beta_S^i)^{n-|H|}-1)\cdot u'(\beta_i)\cdot\beta_i' \\
+ \quad & f\cdot(1-\beta_S^i)^{n-|H|-1}\cdot u(\beta_i) \\
\Longleftrightarrow \quad & \beta_i' \cdot \Bigg[\overbrace{(1+f\cdot(-1+[((1-\beta_S^i)^{n-|H|}-1)\cdot\beta_i+1]))\cdot u''(\beta_i)}^{<0} \\
+ \quad & \overbrace{u'(\beta_i)\cdot f\cdot((1-\beta_S^i)^{n-|H|}-1)}^{<0} \\
+ \quad & \overbrace{u'(\beta_i)\cdot f\cdot((1-\beta_S^i)^{n-|H|}-1)}^{<0} \Bigg] \\
= \quad & \overbrace{u'(\beta_i)\cdot f\cdot\beta_i(1-\beta_S^i)^{n-|H|-1}}^{>0} \\
+ \quad & \overbrace{f\cdot(1-\beta_S^i)^{n-|H|-1}\cdot u(\beta_i)}^{>0} \\
\Rightarrow \quad & \beta_i' < 0
\end{aligned}
$$

References

1. Friesema, I. *et al.* Internet-based monitoring of influenza-like illness in the general population: experience of five influenza seasons in The Netherlands. *Vaccine* **27**, 6353–6357 (2009).
2. Tilston, N. L., Eames, K. T., Paolotti, D., Ealden, T. & Edmunds, W. J. Internet-based surveillance of influenza-like-illness in the UK during the 2009 H1N1 influenza pandemic. *BMC Public Heal.* **10**, 650 (2010).
3. Fleming, D. & Elliot, A. Lessons from 40 years' surveillance of influenza in England and Wales. *Epidemiol. & Infect.* **136**, 866–875 (2008).
4. Elliot, A. J. *et al.* Monitoring the emergence of community transmission of influenza A/H1N1 2009 in England: a cross sectional opportunistic survey of self sampled telephone callers to NHS Direct. *BMJ* **339**, b3403 (2009).
5. Brooks-Pollock, E., Tilston, N., Edmunds, W. J. & Eames, K. T. Using an online survey of healthcare-seeking behaviour to estimate the magnitude and severity of the 2009 H1N1v influenza epidemic in England. *BMC Infect. Dis.* **11**, 68 (2011).
6. Adler, A. J., Eames, K. T., Funk, S. & Edmunds, W. J. Incidence and risk factors for influenza-like-illness in the UK: online surveillance using Flusurvey. *BMC Infect. Dis.* **14**, 232 (2014).
7. Eames, K. *et al.* Rapid assessment of influenza vaccine effectiveness: analysis of an internet-based cohort. *Epidemiol. & Infect.* **140**, 1309–1315 (2012).
8. Peppa, M., Edmunds, W. J. & Funk, S. Disease severity determines health-seeking behaviour amongst individuals with influenza-like illness in an internet-based cohort. *BMC Infect. Dis.* **17**, 238 (2017).
9. Eames, K., Tilston, N. L., Brooks-Pollock, E. & Edmunds, W. J. Measured dynamic social contact patterns explain the spread of H1N1v influenza. *PLOS Comput. Biol.* **8**, 1–8, DOI: 10.1371/journal.pcbi.1002425 (2012).
10. Newman, M. E. J. & Barkema, G. T. *Monte Carlo Methods in Statistical Physics* (Oxford University Press, Oxford, U.K., 1999).
